# Measuring Scientific Reasoning for Graduate Admissions in Psychology and Related Disciplines

**DOI:** 10.3390/jintelligence5030029

**Published:** 2017-07-17

**Authors:** Robert J. Sternberg, Karin Sternberg

**Affiliations:** Department of Human Development, Cornell University, Ithaca, NY 14853, USA; karin.sternberg@gmail.com

**Keywords:** scientific reasoning, graduate admissions, generating hypotheses, generating experiments, drawing conclusions

## Abstract

In two studies, we examined the convergent and discriminant validation of a new assessment of scientific reasoning that could be used for graduate admissions in psychology, educational psychology, human development, and in the psychological sciences more generally. The full assessment ultimately consisted of tests of generating hypotheses, generating experiments, drawing conclusions, serving as a reviewer of a scientific article, and serving as an editor of a scientific journal. The tests had generally good convergent-discriminant validity. Certain socially defined ethnic/racial group differences were observed.

## 1. Introduction

Graduate admissions in psychology-related fields, as well as many other fields, is heavily dependent on use of the Graduate Record Examination (GRE) [1]. Tests such as the GRE, however, measure a relatively narrow band of analytical thinking skills [2,3,4,5]. Is it possible to expand measures to encompass a broader range of skills that might be relevant to graduate and later career success?

Much of the demand in graduate school is on students’ scientific reasoning skills—for example, generating hypotheses, generating experiments, and drawing conclusions from data. The goal of the present research project was to create a test of skill in scientific reasoning that could be used for graduate admissions in psychology or other behavioral and brain sciences.

Our test is based on Sternberg’s [6,7,8,9] theory of successful intelligence. The theory holds that intelligence can be understood in terms of information-processing components that combine to product intelligent behavior.

Metacomponents, or higher-order executive processes, are used to plan what one is going to do, monitor it while it is being done, and evaluate it after it is done. An example of a metacomponent would be formulating a strategy to solve a problem, such as how to design an experiment to test a particular hypothesis.

Performance components are used to execute the strategies formulated by the metacomponents. An example would be inferring relations, as when one infers how the results of a particular test of statistical significance should be interpreted. Knowledge-acquisition components are used to learn how to solve the problem in the first place. An example would be selective comparison, where one tries to ascertain what information stored in long-term memory is relevant to solving a problem, such as retrieving knowledge about how to do an appropriate test of statistical significance in a particular experiment.

The various components can be used in problem-solving in a variety of ways. When they are applied to fairly abstract but relatively unfamiliar kinds of material, they are used analytically. When they are applied to relatively novel tasks or situations, they are applied creatively. And when they are applied to concrete everyday situations, they are applied practically.

According to the theory, individuals can be strong in general abstract analytical skills but not necessarily strong in applying those skills to any one particular domain of practice. That is, analytical intelligence for relatively abstract kinds of problem is largely distinct from the practical intelligence that applies cognitive skills to particular domains of practice. For example, someone might be adept at solving number or letter series, or at solving general mathematical problems, but not be adept when applying the same inductive reasoning skills to a domain of practice such as legal, medical, or scientific problem solving [7,10,11]. The basic argument as it applies here is that the cognitive skills needed to succeed in actual scientific research are in part different from the abstract analytical skills measured by tests such as the GRE. In particular, based on past research (see e.g., [5,12,13,14,15,16,17]), we expected our measures of scientific reasoning to yield scores that are statistically weakly related to scores on tests of academic ability, such as the SAT (formerly the Scholastic Aptitude Test but now named only by acronym), GRE (Graduate Record Examination), or any conventional psychometric test of intelligence.

Sternberg and Williams [17] examined the validity for graduate performance in the Yale Psychology Department of the GRE for predicting (a) first-and second-year course grades, (b) professors’ ratings of student dissertations, and (c) professors’ ratings of students’ analytical, creative, practical, and teaching abilities. The test was found to be predictive only of first-year graduate grades, except that the analytical section was found to be predictive of other criteria, but only for men. In particular, they found the predictive validity of the GRE in predicting first-year grades to be 0.18 for the verbal test, 0.14 for the quantitative test, 0.17 for the analytical test, and 0.37 for the subject-matter test. Sternberg and Williams, however, did not take the next step and propose an alternative test.

Wilson [18] found the predictive validity of the GRE in predicting first-year grades in psychology to be 0.18 for the verbal test, 0.19 for the quantitative test, and 0.32 for the analytical test. Schneider and Briel [19] found the predictive validity of the GRE in predicting first-year graduate psychology grades to be 0.26 for the verbal test, 0.25 for the quantitative test, 0.24 for the analytical test, and 0.36 for the subject-matter test.

Kuncel, Hezlett, and Ones [20] performed a meta-analysis of the predictive validity of the GRE across fields. They found correlations of 0.34 for the GRE verbal, 0.38 for the GRE quantitative, 0.36 for the GRE analytical, and 0.45 for the GRE subject-matter tests. Note that these correlations are corrected for both restriction of range and attenuation. These correlations, therefore, reflect not the correlations for the actual testing, but rather the correlations that in theory would have been obtained for an idealized test of perfect reliability administered to participants with a full range of skill levels. Correlations from the Kuncel et al. study with other criteria were lower, for example, 0.09 for the GRE Verbal, 0.11 for the GRE Quantitative, and 0.21 for the GRE subject-matter test with research productivity (corrected for restriction of range). Correlations with time to complete the degree were 0.28, −0.12, and −0.02, respectively. Kuncel and Hezlett [21] and Kuncel, Wee, Serafin, and Hezlett [22] further found that standardized tests predict graduate students’ success.

A compendium of studies relevant to the preparation and publication of the “new” GRE has been prepared by the Educational Testing Service [23]. The new GRE, like the old GRE, has verbal reasoning and quantitative reasoning sections, but also a writing section. It appears psychometrically to measure constructs similar to those of earlier versions of the test, including general intelligence plus more specific verbal and quantitative knowledge and skills.

The GRE appears to be, at best, a modest predictor of success in graduate school, with its best results for first-year performance, although correlations can be raised by corrections for restriction of range and attenuation (thereby yielding theoretical rather than actual correlations).

The best predictor of future success appears to be the achievement test, suggesting that knowledge of the subject-matter domain may be more important than abstract analytical thinking that is somewhat domain-general and not necessarily highly directly relevant to success in a given academic discipline.

An additional factor to take into account is that data of the Educational Testing Service, which produces the test, shows wide gaps between males and females, on both the verbal and mathematical tests, favoring males [24]. These differences hold up across racial/ethnic groups. But there is no reason to believe that males do better than females in graduate school. The gap in performance therefore is an issue of some concern. There also are racial/ethnic group differences in scores [25]. The existence of such differences might lead one to be at least somewhat concerned about adverse impact in terms of equalizing opportunities for students to pursue graduate education in selective institutions.

New methods of conceptualizing skills for post-baccalaureate training are relevant for business school as well [26,27]. The Graduate Management Admission Test (GMAT) is the most widely used measure of managerial potential in MBA admissions. GMAT scores, although predictive of grades in business school, leave much of the variance in graduate business-school performance unexplained. The GMAT also produces disparities in test scores between groups, generating the potential for adverse impact in the admissions process. Hedlund and colleagues sought to compensate for these limitations by adding measures of practical intelligence to the admissions process in an MBA program. They developed two situational-judgment-test (SJT) approaches to measuring practical intelligence [28,29], a short form with relatively simple problems and a long form with relatively complex problems.

Hedlund et al. [26] administered the resulting measures to two samples of incoming MBA students (total *N* = 792). Across the two studies, they found that scores on both measures predicted success inside and outside the classroom and provided small, yet significant, increments beyond GMAT scores and undergraduate GPA in the prediction of variance in MBA performance. They further found that these measures exhibited less disparity across gender and racial/ethnic groups than did the GMAT.

In particular, the researchers first performed correlational analyses to determine the predictive validity of practical-intelligence scores relative to other predictors and the various performance criteria. Scores on both practical-intelligence measures were predictive of academic success. Students with higher scores on the short-form items had significantly higher first-year and final GPAs (*r* = 0.18 and 0.21, respectively), and also received higher grades on the team-consulting project (*r* = 0.17). Similarly, students with higher scores on the long-form items had significantly higher 1st year and final GPAs (*r* = 0.21 and 0.30, respectively) and higher consulting project grades (*r* = 0.17).

Both GMAT scores and undergraduate GPA also were significant predictors of first-year GPA (*r* = 0.44 and 0.30, respectively) and final GPA (*r* = 0.40 and 0.32, respectively). However, GMAT scores did not correlate significantly with the consulting project grade (*r* = 0.06, ns). Prior work experience did not correlate with MBA grades, and actually exhibited modest negative correlations with practical-intelligence measures (*r* = 0.12) and undergraduate GPA (*r* = 0.22).

Short- and long-form practical intelligence scores exhibited modest, but significant correlations with involvement in extracurricular activities, for which success typically requires some measure of practical intelligence with regard to relating to other people. Students who scored higher on the short-form scores participated in more student clubs (*r* = 0.15) and held more leadership positions (*r* = 0.11). Students with higher long-form scores held more leadership positions (*r* = 0.18).

Situational judgment tasks also can be useful in medical-school admissions. Lievens, Buyse, and Sackett [30] developed an SJT as a possible supplement to cognitive predictors for predicting success in a medical and dental curriculum in Belgium. They found that traditional cognitive predictors were useful, but also that a video-based SJT added significantly to prediction of performance in courses that involved interpersonal aspects of patient care, but not to other courses. Lievens and Sackett [31] subsequently showed that SJT performance was relevant to predicting quality of performance in later internship and professional practice (see also Sternberg [32]).

Shultz and Zedeck [33,34] explored predictors of success in law school beyond the Law School Admission Test (LSAT). They used a broad battery of assessments measuring personality constructs, interests, values, and judgment. Their assessments predicted competency in accomplishing the tasks of a lawyer but at the same time had virtually no adverse impact on underrepresented minority applicants. The authors suggested that their measures, combined with the LSAT and undergraduate GPA, could assess law-school applicants for their predicted professional competence as well as academic performance in law school.

Approaches that go beyond conventional standardized testing have also been proposed at the undergraduate level and below. For example, Oswald, Schmitt, Kim, Ramsay, and Gillespie [35] (see also Schmitt et al. [36]) have found biographical data and situational-judgment tests (the latter of which we also used) to provide incremental validity to the SAT. Sedlacek [37] has developed non-cognitive measures that appear to have had success in enhancing the university-admissions process.

Sternberg and his colleagues (see [5,14,38,39,40,41,42,43,44,45,46,47]) have proposed measures that assess analytical, creative, practical, and wise thinking for undergraduate admissions purposes. They found that they could improve prediction of academic and extracurricular performance in the first year of college and at the same time substantially decrease ethnic-group differences on their assessments (see also Kaufman [48] for the use of creativity measures to reduce ethnic bias).

Stemler, Grigorenko, Jarvin, and Sternberg [49] and Stemler, Sternberg, Grigorenko, Jarvin, and Sharpes [50] found that including creative and practical items in augmented physics, psychology, and statistics AP (Advanced Placement) Examinations reduced ethnic-group differences on augmented test scores relative to the original tests. For other new approaches to college admissions, see Sternberg, Gabora, and Bonney [51].

Grigorenko et al. [52] found that it was possible to improve prediction of private high school (prep school) performance beyond scores attained on the SSAT (Secondary School Admissions Test). And the same principles have been employed in a test for identification of gifted students in elementary school [53].

These findings, although preliminary, suggest the potential value of considering a broader range of abilities in admissions testing at the graduate—and other levels. But how would such tests be constructed? Our rationale in the present research was that it should be possible to create better predictive assessments of graduate (and later) success by measuring analytical reasoning as practiced within the discipline, that is, scientific reasoning as practiced within the field of psychological science. That said, our study is not predictive but rather a correlational study that seeks to explore the potential usefulness of a new assessment for practical scientific reasoning skills relevant to success in psychological sciences.

Based on the theory of successful intelligence, such assessments within a broad-scope scientific reasoning test should correlate with each other (convergent validity) but not correlate as much with tests of abstract analytical reasoning (i.e., *g*) (discriminant validity). Such tests should also have higher content validity and face validity than do the GRE verbal, quantitative, and analytical tests.

The purpose of this report is to describe the results for two studies developing an assessment for graduate admissions purposes. Our basic prediction was that our multiple scientific reasoning assessments would be positively and at least moderately correlated with each other (convergent validity) but only weakly correlated with the measures of fluid and crystallized intelligence as well as with the SAT, which correlates highly with tests of intelligence [54].

The convergent measures were chosen based on the processes of psychological-scientific reasoning presented in virtually all introductory-psychology textbooks as well as experimental methods books. (The reasoning is almost certainly relevant to other sciences as well.) For example, Breedlove [55] describes three of these processes as “come up with alternative hypotheses,” “design an experiment,” and “see which outcome you get, and therefore which hypotheses survived” (p. 41). Although in different texts the exact wording is different, in virtually all of the texts, including our own [56], three of the processes correspond to what we call in our studies “generating hypotheses,” “generating experiments,” and “drawing conclusions.” In terms of the theory of successful intelligence, generating hypotheses and generating experiments fall primarily into the creative domain and drawing conclusions into the analytical domain, but as applied to psychological-scientific research, they also fall into the practical domain—specifically, the practice of doing science. Thus, the use of these analytical and creative skills is contextualized to the psychological-science research domain.

## 2. Study 1

### 2.1. Methods

#### 2.1.1. Subjects

A total of 124 undergraduate students enrolled at Cornell University participated in the data collection. In all, 42 of them were male, and 81 of them were female, with one person not indicating his or her gender. In this sample, 40% of the participants were of European American descent, 34% were of Asian American descent, 6% of African American descent, and 6% of Hispanic American descent. In all, 8% were of other descent and 6% were of mixed descent. Students were enrolled in 38 different majors, with 14 majoring in computer science, 12 majoring in biology and human development each, and 10 majoring in psychology. Participants’ average age was 20.8 years, with a standard deviation of 2.23.

#### 2.1.2. Materials

We used three kinds of assessments—psychometric assessments measuring aspects of psychometrically defined general intelligence, our own scientific reasoning tasks, and a demographic questionnaire. The psychometric tests were chosen to provide strong measures of discriminant validity for our measures. If our measures are assessing nothing more than general intelligence, then there is no need for them, as such tests already exist and are readily available. In particular, we used tests that would tap both fluid abilities and crystallized intelligence.

**Psychometric assessments.** We sought to measure both fluid and crystallized abilities, along the lines suggested by the theories of Carroll [57] and the CHC model [58]. Subjects were presented with a questionnaire that assessed their analytical (fluid) abilities by means of three subtests: (a) a letter-sets test, in which they had to choose a set of letters that did not fit in with other sets of letters presented, (b) a number-series test, in which they had to find the correct number to continue Number Series presented to them, and (c) Miller Analogies Test-type items, in which they had to choose a word out of four options that best completed a given analogy. The Miller-type items measured crystallized as well as fluid abilities. We created the first two tests ourselves, and the third one was taken from a book preparing students for taking the Miller Analogies test [59]. The first two tests are largely measures of fluid intelligence and the last is a measure of both fluid and crystallized intelligence, the latter because much of the difficulty of the items is in knowing the meanings of the words in the analogies (see [57,58,60]).

The main assessments were of scientific reasoning. These tasks were chosen because they represent thinking processes—generating hypotheses, generating experiments, drawing conclusions—that are necessary (although certainly not sufficient) for scientific research [55]. Tasks were informally piloted on small samples of students before use in our research to help select items and set time limits.

**Generating hypotheses.** Subjects were presented with short descriptions of situations and had to create alternative hypotheses to explain the behavior described in the vignettes (see Appendix A for a complete set of vignettes. One vignette said, for example:

Marie is interested in child development. One day, she notices that whenever Laura’s nanny comes in to pick up Laura from nursery school, Laura starts to cry. Marie reflects upon how sad it is that Laura has a poor relationship with her nanny.

What are some alternative hypotheses regarding why Laura starts to cry when she is picked up from nursery school by the nanny?

**Generating experiments.** A second set of vignettes described a situation with hypotheses, and students were asked to design an experiment to test these hypotheses (see Appendix B for a complete set of vignettes. Here is an example:

Ella, a senior in college, observes that her roommate tends to perform better on an exam if she has had a cup of coffee beforehand. Ella hypothesizes that drinking coffee before taking an exam will significantly increase one’s exam performance. However, Ella does not know how to test this hypothesis.

Please suggest an experimental design to test this hypothesis and describe the experiment in some detail. Assume you have the resources you need to be able to do the experiment (e.g., access to students and their academic records, sufficient funds to pay subjects, etc.).

**Drawing conclusions.** A third set of vignettes presented students with the results of studies and asked whether the conclusions drawn were valid (and if not, why not; see Appendix C for a complete set of vignettes. Items looked like the following:

Bill was interested in how well a new program for improving mathematical performance worked. He gave 200 students a pretest on their mathematical knowledge and skills. He then administered the new program to them. After administering the program, he gave the same 200 students a posttest that was equal in difficulty and in all relevant ways comparable to the pretest. He found that students improved significantly in performance from pretest to posttest. He concluded that the program for improving mathematical performance was effective.

**Is this conclusion correct? Why or why not?**

**Demographic questionnaire.** A demographic questionnaire assessed variables like gender, age, major, race, and SAT scores as well as GPAs.

Participants received $20 or course credit for participating in the study. The session lasted approximately one hour.

#### 2.1.3. Design

The design of the study was correlational. In particular, we were interested in latent principal components and common factors that might underlie the observable variables we used.

#### 2.1.4. Procedure

Subjects participated in the study in groups of up to 12 subjects. First, they read and signed an informed-consent form. Then, they were handed out questionnaires. The assessments were arranged in the questionnaire in the following order: Letter Sets test, Number Series test, simulated Miller Analogies test, generating hypotheses, generating experiments, drawing conclusions, demographic questionnaire. The first three tests were timed, so the students were guided together through those three assessments by an experimenter with a stopwatch. After completion of the simulated Miller Analogies test, subjects were allowed to proceed through the materials at their own pace. When they had completed the questionnaire, they returned it to the experimenter, who presented them with a debriefing form. Students read the form and indicated with a signature whether or not their data could be used in our data analysis. Then they received compensation for their participation in the study in the form of either course credit or payment ($20).

### 2.2. Results

#### 2.2.1. Basic Statistics

Table 1 shows the mean scores and standard deviations on scales used in the study.

The total scores on the simulated Miller Analogies were low (with a mean of 4.80 out of 21 and a standard deviation that was almost ¾ of the value of the mean), suggesting that, however difficult (or valid) the Miller Analogies test might be for other populations, our simulated test was simply too difficult for our subjects to be viable. We therefore excluded it from the principal component and common factor analyses presented below. (However, when it was included, it factored with the letter—and number-series tests.) We decided not to use this test in Study 2 of the project.

Respective means for men versus women were 7.25 (SD = 2.94) versus 8.27 (SD = 2.88) for generating hypotheses, 6.83 (SD = 1.40) versus 7.13 (SD = 1.85) for generating experiments, and 6.45 (SD = 1.44) versus 6.92 (SD = 1.75) for drawing conclusions. Sex differences were tested via a multivariate linear model. Overall degrees of hypothesis (gender) degrees of freedom were (3, 117). The overall multivariate *F* for sex differences was *F* (3, 117) = 1.47 (Pillai’s trace = 0.036), which was not significant (*p* = 0.226). Because the overall *F* was not significant, no follow-ups are reported.

There were two socially-defined racial/ethnic groups for which we had sufficient data to draw comparisons: European Americans and Asian Americans. Respective means for Asian Americans versus European Americans were 7.23 (SD = 2.64) and 8.54 (SD = 2.88) for generating hypotheses, 7.23 (SD = 1.57) and 7.13 (SD = 1.91) for generating experiments, and 6.63 (SD = 1.56) and 7.26 (SD =1.72) for drawing conclusions. Socially-defined racial/ethnic group differences were tested via a multivariate linear model. With general linear-model testing, the overall multivariate *F* (3, 86) for socially defined racial/ethnic group was 2.32 (*p* = 0.081) (Pillai’s trace = 0.075), which was not statistically significant (*p* = 0.081). Because the overall *F* was not significant, no follow-ups are reported.

#### 2.2.2. Interrater Reliability

Ratings took into account quality of responses and, where appropriate, quantity (for the assessment on generating hypotheses). Quality was judged on the basis of principles of experimental design, such as having one or more appropriate control groups, having an experiment that actually tests a proposed hypothesis, not having confounded variables, and proposing a design that would lend itself to statistical analysis. For quantity, responses that were minor variants of each other were not counted separately in quantity ratings; further, responses that were incorrect (e.g., hypotheses not relevant to the stated problem) or that were inappropriate to the problem as presented were not counted in quantity ratings.

Interrater reliability for two independent raters was computed by correlating scale sum scores of Raters 1 and 2. Interrater reliability was 0.75 for the “drawing conclusions” subscale, 0.96 for the “generating hypotheses” subscale, and 0.78 for the “generating experiments” subscale. When one total score over all three scales was created for each rater, interrater reliability was 0.92. When scoring the “generating hypotheses” subscale, the scoring process consisted solely of counting valid hypotheses. For this reason, the interrater reliability of this subscale is particularly high. These reliabilities suggest that the qualities we are measuring can be assessed in a reliable fashion.

#### 2.2.3. Intercorrelations

Table 2 shows the intercorrelation matrix for the variables in our study. Our three subscales (hypotheses, experiments, and conclusions) did not significantly correlate with any of the scales assessing analytical abilities. There was also no significant correlation of our subscales with Cornell GPA. Our assessment showed significant negative correlations of the hypotheses and conclusions scales with the SAT math and reading scores. Consistent with our hypotheses, these data suggest that our scales are assessing an ability construct that is different from (or possibly even inverse to) the abilities assessed by SAT and academic GPA.

#### 2.2.4. Principal Components and Common Factor Analyses

We did both principal components and common factor analyses. We did exploratory principal components analyses because the studies here are exploratory—first-time evaluations of potential graduate admissions tests we have newly proposed. We believe that the principal component results are most appropriate for our initial exploratory purposes. However, we also did maximum likelihood common factor analysis, which yielded similar but somewhat less clear-cut results. For the sake of completeness, we present both sets of results in each case. We shall draw our interpretations based primarily on the principal components analyses.

The tables below show the results of exploratory analyses with Varimax rotation, including: our scales and the analytical ability scales (Table 3 and Table 4), our scales and students’ SAT scores (Table 5 and Table 6), or all of the scales (our scales, SAT scores, and the analytical ability scales) (Table 7 and Table 8).

The results show that our new subscales load on a different principal component from either analytical ability subscales or SAT scores. In each case, our assessments loaded on one principal component (either the first or the second) and the psychometric assessments loaded on the other principal component.

### 2.3. Discussion

In summary, the scales in Study 1 appear to have assessed interrelated research skills not assessed by the SAT or scales that measure analytical intelligence and common academic knowledge. Our tests form one clear principal component and the analytical ability tests combined with the SAT form another. Our tests, if anything, showed negative correlations with the SAT. Restriction of range as a result of using Cornell students as subjects might have been expected to reduce magnitudes of correlations. The results, then, seem promising.

Nevertheless, the tasks we used are fairly simple as research-based tasks go. In Study 2, we decided to use more complex tasks.

## 3. Study 2

In Study 2, we decided to use two tasks that are seemingly more complex than those used in Study 1, because they involved not just single acts (e.g., generating hypotheses) but rather the multiple acts involved in reviewing articles and reviewing reviews of articles. Specifically, we used tasks requiring subjects to act as simulated reviewers for journals and as simulated editors.

### 3.1. Methods

#### 3.1.1. Subjects

A total of 149 undergraduate students enrolled at Cornell University participated in the data collection. In all, 42 of them were male and 107 of them were female. In this sample, 43% of the participants were of European American descent, 34% were of Asian American descent, 6% were of African American descent, and 11% were of Hispanic American descent. In addition, 4% were of other descent and 2% were of mixed descent. Students were enrolled in 41 different majors, with 11 majoring in human development, 18 majoring in psychology, 17 majoring in biology, and 22 majoring in Biology and Society. Participants’ average age was 20.2 years, with a standard deviation of 2.28.

#### 3.1.2. Materials

Subjects were presented with a questionnaire that assessed their analytical abilities in two subtests: (a) a letter-sets test, in which they had to choose of set of letters that did not fit in with other sets of letters presented, and (b) a number-series test, in which they had to find the correct number to continue number series presented to them. The items were created by us and were the same ones we used in Study 1.

Next, subjects worked on a series of items that were newly developed for Study 2. These items were of two kinds.

**Reviewer.** In this item type, comprising two items, students had to play the role of a reviewer of a journal article. They read a two-page description of an experiment and then were asked to indicate the article’s flaws and problems. Answers were given in the style of an essay (writers could write whatever they wanted), much as would be a review of a journal article written for a journal editor. In one of the items, participants read a short report on the validation of an extraversion scale. After reading the report, they were asked: “Is the article you just read adequate or not? If not, what are the article’s flaws and problems?” Participants answered in essay style. For more information, please see Appendix D.

**Editor.** In this item type, comprising a single item, participants were placed in the role of a journal editor who had to judge a review of an article. Students were presented with 13 statements about an article (which they had not read) and had to evaluate each statement. Answers were again given in free text format (i.e., subjects could write whatever they wanted), but this time the answers consisted mostly of a sentence or two judging the adequacy of each review statement. Sample statements the participants had to comment on are:

- Another problem with the study is that the authors did not randomly assign students either to age groups or to attachment styles, so random assignment, the gold standard for experimental research, was lacking.
- The study was conducted only with subjects in the Northeast, so it is not clear whether the results would generalize to other parts of the country or to other countries.
  For more information, please see Appendix E.

**Tasks from Study 1.** The following section comprised the three subtests we had created for Study 1: generating hypotheses to explain behaviors described in vignettes, generating experiments to test hypotheses described in vignettes, and drawing conclusions from results presented in vignettes (see Study 1 above).

A demographic questionnaire assessed variables including gender, age, major, race, and SAT/ACT/GRE scores as well as GPA scores. Cole and Gonyea [61] reported correlations between actual and self-reported scores to be high, although higher for higher scorers than for lower scorers. At Cornell, most students are relatively high scorers, so these data, although not perfect, can be expected to be at least an approximation to the actual numbers. Gender and racial/ethnic identification were included because previous research has found differences in standardized cognitive-test scores across gender and racial/ethnic groups (e.g., [24,25]). We also asked students to provide information about their prior research experience, how many lab courses or classes on research methods they had taken, and how many scientific articles they read per month. Participants received $20 or course credit for participating in the study, which lasted roughly 1.5 h.

#### 3.1.3. Design

The design of the study was correlational. In particular, we were interested in latent principal components and common factors that might underlie the observable variables we used.

#### 3.1.4. Procedure

Subjects participated in the study in groups of up to 12. First, they read and signed an informed-consent form. Then, they received the questionnaires. The assessments were arranged in the questionnaire in the following order: letter-sets test, number-series test, reviewer items, editor items, generating hypotheses, generating experiments, drawing conclusions, demographic questionnaire. The first two tests were timed, so the students were guided through those two assessments together by the experimenter with a stopwatch. Afterwards, subjects were allowed to proceed at their own pace. Students were allowed to withdraw from the study at any time. When they had completed the questionnaire, they returned it to the experimenter, who presented them with a debriefing form. Then they received compensation for their participation in the study in the form of either credits or payment ($20).

### 3.2. Results

#### 3.2.1. Basic Statistics

Table 9 shows the mean scores and standard deviations on scales used in the study. All of the tasks showed satisfactory means and standard deviations.

Means by sex for men versus women were 0.7.60 (SD = 2.61) and 8.07 (SD = 2.64) for generating hypotheses, 6.26 (SD = 1.37) and 6.62 (SD = 1.58) for generating experiments, 6.38 (SD = 1.12) and 6.34 (SD = 1.32) for drawing conclusions, 5.62 (SD = 3.02) and 7.85 (SD = 5.12) for reviewer, and 17.47 (SD = 4.62) and 16.67 (SD = 5.06) for editor. The overall hypothesis multivariate effect for gender was tested at *F* (5, 101) = 1.76 (Pillai’s trace = 0.08). As the overall *F* was not significant (*p* = 0.129), follow-up tests of statistical significance are not presented.

The only two groups for which there were sufficient subjects to conduct tests of statistical significance were Asian Americans and European Americans. Respective Means for socially-defined racial/ethnic groups for Asian-American and European-Americans were 7.26 (SD = 2.78) and 8.48 (SD = 2.39) for generating hypotheses, 6.01 (SD = 1.28) and 6.92 (SD = 1.59) for generating experiments, 6.19 (SD = 1.36) and 6.48 (SD = 1.18) for drawing conclusions, 6.54 (SD = 4.00) and 7.79 (SD = 5.21) for reviewer, and 15.59 (SD = 3.97) and 17.90 (SD = 5.39) for editor. The overall multivariate effect was tested using the general linear model at *F* (5, 101) = 3.04, *p* = 0.013 (Pillai’s trace = 0.131). Because the overall multivariate *F* was statistically significant (*p* = 0.013), we followed up with univariate analyses of variance. The resulting *F’s*, each consuming one degree of freedom, were 6.02 (*p* = 0.016) for generating hypotheses, 10.08 (*p* < 0.002) for generating experiments, 1.33 (not significant) for drawing conclusions, 1.85 (not significant) for reviewer, and 6.08 (*p* = 0.015) for editor. All the differences were in favor of the European-American group. These differences were not predicted and it is hard to know what to make of them.

#### 3.2.2. Interrater Reliability

Ratings took into account quality of responses and, where appropriate, quantity (for the assessment on generating hypotheses). Interrater reliability for two independent raters was computed by correlating scale sum scores of Raters 1 and 2 using Pearson product-moment correlations [62]. Interrater reliability was 0.67 for the “drawing conclusions” subscale, 0.97 for the “generating hypotheses” subscale (which was basically a matter of counting valid hypotheses), and 0.81 for the “generating experiments” subscale. When one total score over all three scales was created for each rater, interrater reliability was 0.91. The interrater reliability for the Reviewer items was 0.97 and for the Editor items it was 0.83. (The high reliability of the Reviewer items reflects in part the fact that the task of the scorers was primarily to count valid responses.) These reliabilities suggest that the qualities we are measuring can be assessed in a reliable fashion.

#### 3.2.3. Intercorrelations

Table 10 shows the intercorrelation matrix for the variables in the study.

None of our scales correlated significantly with the number of lab courses a student has taken. However, all of our scales except the “generating experiments” scale correlated significantly with the number of journal articles a student reads per month.

In Study 2, all of our five subscales significantly correlated with the letter-sets test (as shown in the table—editor, reviewer, hypotheses, experiments, conclusions: 0.19, 22, 0.26, 0.18, 34) and the Editor and Conclusions scales correlated significantly with the Number Series test (0.33, 0.17). This is in contrast to Study 1, where we found almost no significant correlations of our scales with the analytical ability tests. However, the median correlation for the first group was 0.22 and for the second group was 0.25, so the correlations were relatively weak.

There were no significant correlations of Cornell GPA with four of our subscales (Hypotheses, Experiments, Reviewer and Editor). The conclusions scale did correlate significantly with GPA (0.25), however. As expected, SAT math scores did not significantly correlate with any of our five subscales, and the SAT reading scores significantly correlated only with the Editor (0.23) and Hypotheses (0.25) items. Again, these are relatively weak correlations, especially in view of the high inter-rater reliability of our assessments.

The data generally are consistent with our hypothesis that our scales largely assess a construct different from GPA, SAT, and psychometric tests of inductive-reasoning ability. The data were consistent with our hypotheses, although somewhat weaker than in Study 1. In particular, the editor items have proven not to be the most promising assessment. They are unfamiliar in scope for our subjects and thus ended up being in large part a measure of fluid ability.

#### 3.2.4. Principal Component and Common Factor Analyses

We replicated the principal component and common factor analyses we did in Study 1 to see whether we could replicate the results. As can be seen in Table 11, Table 12, Table 13, Table 14, Table 15 and Table 16, the Hypotheses, Experiments, and Conclusions items loaded on a separate component from the Letter Sets/Number Series items or the SAT reading/math scores. This is the same result we saw in Study 1. Common factor analyses yielded similar results and conclusions were no different.

When we added both the two SAT scores and the analytical ability scales to a principal component and common factor analysis with the Hypotheses, Experiments, and Conclusions scales, the difference from Study 1 was that now that the SAT scores and analytical ability scales loaded on separate principal components and common factors. Our three scales still loaded on the same component (a different one from the SAT and analytical ability components), but in this analysis, the Conclusions scale also loaded on the principal component of the analytical ability scales.

We then we computed average scores for the SAT scores (consisting of each participant’s SAT math and SAT reading scores) and the analytical ability scales (consisting of the Letter Sets and number-series scores). Table 17, Table 18, Table 19 and Table 20 shows the results of a principal component and common factor analyses with Varimax rotations, including our scales and the averages for the SAT scores and analytical ability scores.

SAT and analytical ability scores clearly load together on one component as expected. The scores of the Reviewer items, generating hypotheses items, and generating experiments items load on a separate principal component. The drawing conclusions scores loaded strongest on the component of our newly created scales but also loaded on the analytical ability scales component. The editor scores also loaded on the analytical ability scales component, suggesting that the editor scale was not as effective as we had hoped. In retrospect, we believe it was too novel and unfamiliar to our subjects and thus became more of a fluid ability test than we had anticipated.

In summary, the scales we have created so far to assess students’ research skills mostly tap into an ability that is not well assessed by the SAT, GRE, or by scales that measure analytical intelligence. The editor items did not seem to work as well as the other items we created.

### 3.3. Discussion

For the present series of studies, we have created a series of items that can be used as a supplemental means for graduate admissions in psychology, human development, and perhaps other fields as well. The items assessed five different but related skills:

- generating hypotheses
- generating experiments
- drawing conclusions
- reviewing scientific articles
- evaluating reviews of scientific articles.

We chose these skills because they are ones that scientists, including but not limited to faculty members, need to succeed in their careers, and because the current graduate admissions instruments do not adequately predict the future success of graduate students once they enter their academic career [1,17].

Study 1 (which used the first three items types mentioned above) was consistent with our hypothesis that we created items that measure a scientifically useful set of skills not captured by GPA, SAT, or analytical ability test scores. None of the three item types we developed correlated significantly positively with measures of analytical ability, and in some cases (in Study 1) they correlated negatively with both SAT math and reading scores. Principal component analyses with Varimax rotation were consistent with our hypotheses further by showing that SAT and analytical ability scores loaded on one component and our new measures loaded on a second component. Common factor analyses showed similar results.

Study 2 used the same three item types and additionally employed two more item types to measure students’ ability to review scientific articles and to evaluate the quality of a review of a scientific article. The data were generally consistent with our hypotheses, although in not quite as strong a way. We found that the editor items did not work as well as we had hoped because, we believe, the task was just too far beyond what students were ready to do. Students likely had at least some experience assessing scientific articles, but probably had no experience serving as journal editors assessing assessments of scientific articles. We thus excluded the Editor items from the following data analyses.

We found that all of the remaining four of our item types (Hypotheses, Experiments, Conclusions, and Reviewer) correlated significantly but modestly with the letter-sets test, and the conclusions items correlated weakly but significantly with the Number Series scale. In Study 2, there were no negative correlations of our items with the SAT scores; rather, the correlations were weakly positive. Only the Generating Hypotheses items correlated significantly and positively with the SAT reading scores. Thus, the results still suggest that our measures assess skills that are distinct from the skills that the SAT measures.

In a principal component analysis with Varimax rotation, we found once again that SAT scores and analytical abilities scores usually loaded on the same component. According to expectations, the reviewer items as well as generating hypotheses and generating experiments items loaded on a separate component. The outlier in the principal component analysis was the drawing conclusions subtest, which loaded about the same on both components. Common factor analyses showed similar results.

The data from two different data collections to assess skills different from those assessed by SAT scores and college GPA are consistent. In particular, they argue for the construct validity of the generating hypotheses and generating experiments items, which worked well in both studies in which they were used. The drawing conclusions items worked very well in the first study but not quite as well in the second study. The Reviewer items worked well in the study in which they were used; the Editor items did not work as well (because, we believe, they were too unfamiliar for the students and thus ended up measuring mainly fluid abilities).

## 4. General Discussion

We have proposed new measures that potentially could be used to assess prospective students’ potential future success in graduate training and in their chosen research careers. The tests measure various aspects of scientific reasoning: generating hypotheses, generating experiments, drawing conclusions, reviewing, and editing. The editing task appears to have been quite novel for our subjects and may thus have tapped into fluid intelligence skills as well as whatever else it is that our scientific reasoning tasks measure. These tests are not viewed as a replacement for existing measures, but rather as a supplement to them [5,14]. Of course, graduate success in psychological science has many aspects: Our focus is on the aspect that we believe is most important—scientific reasoning—that conventional tests used for admission fail to measure directly.

We believe that the data we obtained are promising, and that they are conservative. Our subjects were Cornell university undergraduates, who are well above average with regard to academic skills. Our correlations were not corrected for restriction of range. We could not correct for restriction of range in our correlations because although we know our students were well above average on conventional standardized tests, we have no way of assessing how they differed from a typical psychology graduate applicant population in scientific reasoning skills. Moreover, our scientific reasoning measures did not show the kinds of correlations with conventional admissions measures that might suggest that high scores on conventional measures might lead to high scores on our assessments of scientific reasoning; quite the contrary. Correcting for restriction of range, therefore, might have inflated the correlations. All that said, our subjects were above average for college students and probably for applicants to graduate school, so we cannot be certain how much generality there would be of our results to academically more diverse populations.

The research described here is obviously a beginning, not an end to an attempt to devise measures to supplement standard assessments for measuring cognitive skills needed for success in graduate school and in scientific careers.

First, although we know that the correlations of the assessments with each other are substantial but, in general, with measures of general intelligence are lower, it remains to be seen whether the assessments add prediction to measures of success in graduate school. We have reason to believe that there is more to success in graduate school than what the GRE measures [17], but we do not know for sure that what remains to be predicted is what our assessments measure.

In the ideal case, we would have predictive-validity data for graduate school or career success. In practice, such data are hard but not impossible to obtain. If one uses first-year graduate students as participants, one can follow their careers through graduate school. However, in any reasonably competitive graduate program, the matriculated students are already a severely restricted range of applicants, so any correlations obtained for them will be suspect. It thus may make sense to provide in reports of such data both the original correlations and the correlations corrected for restriction of range, if one knows the range of the relevant population. Moreover, applicants are not at the level of advancement in education one is really interested in, namely, undergraduates who are or will be applying to graduate schools. If one uses instead students and especially seniors in college, obtaining predictive-validity data is difficult. Some of the students ultimately will go to graduate school, others will not, so there will be a (possibly severe) drop-out effect, and one skewed toward those with stronger credentials. Moreover, even those who go to graduate schools will go to different ones, so that it will be hard to obtain data, and even the data obtained will be very difficult to compare from one graduate institution to another.

Second, the assessments we have now measure scientific reasoning skills, but do not measure all the skills relevant to success in graduate school and in careers. For example, we have not included problems requiring reasoning with concepts from probability and statistics. The work of Kahneman and Tversky [63,64] suggests that such reasoning is important in understanding one’s data. Stanovich [65,66] have argued that rational thinking, of the kinds measured by the Kahneman and Tversky problems and others like them, measure a construct that is important but largely missed by conventional standardized tests. Motivation also is important for success, on tests but more importantly in graduate school [67,68,69]. What is arguably an even more important skill for success in graduate school and in a career is creativity or taste in problems [70,71,72]. In the end, creativity in the field is at least as important as scientific reasoning where the problem is a given.

Third, the predictive value of our assessment, like any assessment, will depend on the culture in which it is used [73,74,75]. This assertion applies not only to cultures across nations, but also to departmental cultures. The assessment as it now exists emphasizes research skills. But to have a really good predictive test, one needs a strong sense of exactly what it is that one wants to predict [76]. In a teaching-based institution—perhaps a small college—research skills may matter less and teaching skills more. Current research with an assessment to measure teaching skills will investigate such measures (see [77] in preparation).

Fourth, other theories of intelligence and related constructs might suggest further kinds of assessments to add that would be relevant to prediction of graduate school success in psychology. For example, Carroll’s [57] theory has many abilities potentially relevant to graduate study that are not assessed in conventional measures. Ceci’s [78] bioecological approach might suggest contextual measures relevant to graduate study. Gardner’s [79] theory might suggest additional measures of what he refers to as interpersonal and intrapersonal intelligence. Mayer and Salovey’s [80] concept of emotional intelligence also might be relevant. It is probably overlapping with Gardner’s interpersonal and intrapersonal intelligences and Sternberg’s practical intelligence construct. It remains to be seen what these theories might add in terms of measures.

Fifth, there is today as always the question of whether the kinds of tests we use are equitable across different racial, ethnic, and other groups (e.g., [81,82,83,84]). There are many different views on whether the tests are fair across groups. In our own work, we have found that the kinds of tests we used tend to reduce racial-ethnic differences while increasing prediction [14]. We did not have enough subjects of different racial/ethnic groups to make any kind of determination in the present studies. This issue would remain for further studies to sort out.

Finally, our experience is that it is extremely difficult to get universities to change their admissions procedures [1,5]. There is a great deal of entrenchment and reluctance to change, resulting in admissions systems that remain more or less constant decade after decade.

Nevertheless, psychology departments and departments in related fields (education, human development, cognitive science) probably can do better in selecting those students who will not only or even necessarily get the best grades, but rather who will be the best scientists. A test of scientific reasoning as part of the admissions process seems like a good first step in that direction. Any such test measures only a particular set of skills at a given point of time. One can argue whether general intelligence is modifiable (e.g., Herrnstein and Murray [83], taking a negative position; Feuerstein [85], taking a positive position). However, scientific reasoning skills can certainly be improved [86,87,88,89], at least in part by exercising them and reflecting on how one can make them better. For example, one learns how to review articles by reviewing them and by reading other people’s reviews. So our kind of assessment can be used diagnostically to aid improvement as well as to evaluate mastery of skills. In the end, using assessments for diagnostic purposes to improve skills may be more valuable to the science of psychology than merely using them to test for mastery.

## Figures and Tables

**Table 1:** Mean scores and standard deviations in Study 1.

Measure	Mean	Standard Deviation
Cumulative Cornell GPA	3.48	0.41
SAT Reading Score	681	113
SAT Math Score	720	112
Letter Sets total score	10.25	2.68
Number Series total score	11.49	3.02
Miller Analogies total score	4.80	3.44
Hypotheses total score	7.93	2.91
Experiments total score	7.07	1.77
Conclusions total scores	6.79	1.73

**Table 2:** Intercorrelations in Study 1.

	1	2	3	4	5	6	7	8	9	10	11	12	13	14	15	16	17
1 Hypotheses	1	0.09	0.33 **	0.80 **	0.80 **	−0.01	−0.03	−0.03	−0.04	0.00	0.17	0.15	0.16	−0.00	−0.21 *	−0.24 *	0.14
2 Experiments	0.09	1	0.43 **	0.57 **	0.57 **	0.00	0.01	0.14	0.22 *	0.24 **	0.010	0.10	−0.02	0.05	−0.01	−0.07	−0.09
3 Conclusions	0.33 **	0.43 **	1	0.72 **	0.72 **	0.13	0.145	0.04	0.11	0.17	0.14	0.08	−0.05	0.11	−0.18	−0.24 *	−0.05
4 ExpConcl	0.80 **	0.57 **	0.73 **	1	1.00 **	0.02	0.02	0.04	0.09	0.14	0.19*	0.18*	0.09	0.08	−0.21 *	−0.29 **	0.00
5 HypExpConc	0.80 **	0.57 **	0.73 **	1.00 *	1	0.02	0.02	0.04	0.09	0.14	0.19 *	0.18*	0.09	0.08	−0.21 *	−0.29 **	0.00
6 Letter Sets	−0.01	0.00	0.13	0.02	0.02	1	0.99 **	0.34 **	0.23 **	0.24 **	−0.04	−0.16	−0.23 **	0.13	0.25 *	0.24 *	−0.04
7 LS corr. Ans	−0.03	0.01	0.15	0.02	0.02	0.99 **	1	0.37 **	0.24 **	0.26 **	−0.06	−0.17	−0.24 **	0.15	0.29 **	0.28 **	−0.05
8 NS corr Ans	−0.03	0.14	0.04	0.04	0.04	0.34 **	0.37 **	1	0.12	0.14	−0.39 **	−0.09	−0.10	0.17	0.18	0.44 **	−0.07
9 Total Score MAT	−0.04	0.22 *	0.11	0.09	0.09	0.23 **	0.24 **	0.12	1	0.97 **	−0.10	0.03	−0.06	0.05	0.23 *	0.05	0.01
10 MAT cor ans	0.00	0.24 **	0.17	0.14	0.14	0.24 **	0.25 **	0.14	0.97 **	1	−0.09	0.00	−0.10	0.03	0.24 *	0.03	0.03
11 Gender	0.17	0.10	0.14	0.19 *	0.19 *	−0.04	−0.06	−0.39 **	−0.10	−0.09	1	0.13	0.26 **	−0.01	−0.10	−0.30 **	−0.12
12 Age	0.15	0.10	0.08	0.18 *	0.18 *	−0.16	−0.17	−0.09	0.03	0.01	0.13	1	0.85 **	0.09	−0.34 **	−0.27 **	−0.136
13 Year	0.16	−0.02	−0.05	0.09	0.09	−0.23 **	−0.24 **	−0.10	−0.06	−0.10	0.26 **	0.85 **	1	0.06	−0.28 **	−0.24 *	−0.13
14 Cornell GPA	−0.00	0.05	0.11	0.08	0.08	0.13	0.15	0.17	0.05	0.03	−0.01	0.09	0.06	1	0.08	0.10	−0.03
15 SAT Reading	−0.21 *	−0.01	−0.18	−0.21 *	−0.21 *	0.25 *	0.29 **	0.18	0.23 *	0.24 *	−0.10	−0.34 **	−0.28 **	0.08	1	0.78 **	0.04
16 SAT Math	−0.24 *	−0.07	−0.24 *	−0.29 **	−0.29 **	0.24 *	0.28 **	0.44 **	0.05	0.03	−0.30 **	−0.27 **	−0.24 *	0.10	0.78 **	1	−0.02
17 Ethnicity	0.14	−0.09	−0.05	0.00	0.000	−0.04	−0.05	−0.07	0.01	0.03	−0.12	−0.14	−0.13	−0.03	0.04	−0.02	1

* Correlation is significant at the 0.05 level (two-tailed). ** Correlation is significant at the 0.01 level (two-tailed).

**Table 3:** Rotated principal component matrix in the Study 1 principal components analysis of the total scores of our subscales and the analytical ability tests.

Rotated Component Matrix ^a^		
	Component	
	1	2
Generating Hypotheses total score averaged over both raters	0.64	−0.15
Generating experiments total score averaged over both raters	0.67	0.12
Drawing conclusions total score averaged over both raters	0.84	0.08
Total Score Letter Sets	0.00	0.80
Number Series correct answers	0.04	0.82

Extraction Method: Principal Component Analysis. Rotation Method: Varimax with Kaiser Normalization. ^a^ Rotation converged in three iterations. Two principal components had Eigenvalues greater than 1. Component 1 had an Eigenvalue of 1.56, accounting for 31.1% of the variance in the data. Component 2 had an Eigenvalue of 1.36, accounting for 27.2% of the variance in the data. Cumulative percent variance accounted for was 58%.

**Table 4:** Rotated maximum likelihood common factor matrix in the Study 1 factor analysis of the total scores of our subscales and the analytical ability tests.

Rotated Factor Matrix ^a^		
	Factor	
	1	2
Generating Hypotheses total score averaged over both raters	0.34	−0.02
Generating experiments total score averaged over both raters	0.37	0.15
Drawing conclusions total score averaged over both raters	1.00	0.06
Total Score Letter Sets	0.08	0.76
Number Series correct answers	−0.03	1.00

Extraction Method: Maximum likelihood factor method. Rotation Method: Varimax with Kaiser normalization. ^a^ Rotation converged in three iterations. Factor 1 had an Eigenvalue of 1.26, accounting for 25.2% of the variance in the data. Factor 2 had an Eigenvalue of 1.14, accounting for 22.9% of the variance in the data. Cumulative percent variance accounted for was 48%.

**Table 5:** Rotated principal component matrix in the Study 1 analysis of the total scores of our subscales and SAT scores.

Rotated Component Matrix ^a^		
	Component	
	1	2
Generating Hypotheses total score averaged over both raters	−0.26	0.59
Generating experiments total score averaged over both raters	0.12	0.77
Drawing conclusions total score averaged over both raters	−0.19	0.78
SAT Math Score	0.92	−0.17
SAT Reading Score	0.94	−0.05

Extraction Method: Principal component analysis. Rotation Method: Varimax with Kaiser normalization. ^a^ Rotation converged in three iterations. Two principal components had Eigenvalues greater than 1. Component 1 had an Eigenvalue of 1.83, accounting for 36.7% of the variance in the data. Component 2 had an Eigenvalue of 1.58, accounting for 31.6% of the variance in the data. Cumulative percent variance accounted for was 68%.

**Table 6:** Rotated maximum likelihood common factor matrix in the Study 1 factor analysis of the total scores of our subscales and SAT scores.

Rotated Factor Matrix ^a^		
	Factor	
	1	2
Generating Hypotheses total score averaged over both raters	−0.20	0.39
Generating experiments total score averaged over both raters	0.01	0.45
Drawing conclusions total score averaged over both raters	−0.15	0.80
SAT Math Score	0.80	−0.21
SAT Reading Score	0.97	−0.06

Extraction Method: Maximum likelihood factor method. Rotation Method: Varimax with Kaiser normalization. ^a^ Rotation converged in three iterations. Factor 1 had an Eigenvalue of 1.63, accounting for 32.6% of the variance in the data. Factor 2 had an Eigenvalue of 1.04, accounting for 20.7% of the variance in the data. Cumulative percent variance accounted for was 53%.

**Table 7:** Rotated principal component matrix in the Study 1 analysis of the total scores of our subscales, the analytical ability tests, and SAT scores.

Rotated Component Matrix ^a^		
	Component	
	1	2
Generating Hypotheses total score averaged over both raters	−0.15	0.62
Generating experiments total score averaged over both raters	0.15	0.67
Drawing conclusions total score averaged over both raters	−0.06	0.80
SAT Math Score	0.83	−0.35
SAT Reading Score	0.75	−0.31
Total Score Letter Sets	0.58	0.10
Number Series correct answers	0.70	0.21

Extraction Method: Principal component analysis. Rotation Method: Varimax with Kaiser normalization. ^a^ Rotation converged in three iterations. Two principal components had Eigenvalues greater than 1. Component 1 had an Eigenvalue of 2.13, accounting for 30.4% of the variance in the data. Component 2 had an Eigenvalue of 1.75, accounting for 25.0% of the variance in the data. Cumulative percent variance accounted for was 55%.

**Table 8:** Rotated maximum likelihood common factor matrix in the Study 1 factor analysis of the total scores of our subscales, the analytical ability tests, and SAT scores.

Rotated Factor Matrix ^a^		
	Factor	
	1	2
Generating Hypotheses total score averaged over both raters	−0.10	0.42
Generating experiments total score averaged over both raters	0.06	0.47
Drawing conclusions total score averaged over both raters	−0.04	0.76
SAT Math Score	0.94	−0.33
SAT Reading Score	0.75	−0.23
Total Score Letter Sets	0.26	0.04
Number Series correct answers	0.49	0.10

Extraction Method: Maximum likelihood method. Rotation Method: Varimax with Kaiser normalization. ^a^ Rotation converged in three iterations. Factor 1 had an Eigenvalue of 1.78, accounting for 25.4% of the variance in the data. Factor 2 had an Eigenvalue of 1.15, accounting for 16.4% of the variance in the data. Cumulative percent variance accounted for was 42%.

**Table 9:** Mean scores and standard deviations in Study 2.

Measure	Mean	Standard Deviation
Cumulative Cornell GPA	3.41	0.41
Reported SAT Reading score	694	84
Reported SAT Math score	712	88
Letter Sets total score	10.05	2.38
Number Series total score	10.57	2.96
Hypotheses total score	7.95	2.66
Experiments total score	6.52	1.52
Conclusions total scores	6.29	1.25
Reviewer total score	7.32	4.58
Editor total score	16.87	4.90

**Table 10:** Intercorrelations in Study 2.

		1	2	3	4	5	7	8	9	10	11	12	13	14	15	16	17
1	Editor	1	0.40 **	0.18 *	0.27 **	0.22 **	0.11	0.23 *	0.12	−0.14	0.08	0.17 *	0.17 *	0.08	0.20 *	0.21 *	0.33 **
2	Reviewer	0.40 **	1	0.38 **	0.29 **	0.24 **	0.06	0.17	0.06	0.21 *	−0.01	0.15	0.08	0.13	0.23 **	0.23 **	−0.00
3	Hypotheses	0.18 *	0.38 **	1	0.34 **	0.30 **	0.03	0.25 **	0.12	−0.02	−0.15	−0.09	0.13	0.01	0.17 *	0.28 **	0.04
4	Experiments	0.27 **	0.29 **	0.34 **	1	0.36 **	−0.12	0.17	0.05	0.09	−0.05	0.02	0.12	−0.02	0.15	0.18 *	0.08
5	Conclusions	0.22 **	0.24 **	0.30 **	0.36 **	1	0.25 **	0.11	0.15	−0.06	−0.09	−0.04	0.03	0.16	0.22 **	0.36 **	0.17 *
7	Cornell GPA	0.11	0.06	0.03	−0.12	0.25 **	1	0.045	0.08	−0.09	0.11	0.09	0.01	−0.12	−0.04	0.06	0.21 *
8	SAT Reading	0.23 *	0.17	0.25 **	0.17	0.11	0.05	1	0.67 **	0.01	−0.08	−0.06	−0.01	−0.11	0.03	0.16	0.20 *
9	SAT Math	0.12	0.06	0.12	0.05	0.15	0.08	0.67 **	1	−0.21 *	−0.05	−0.00	−0.04	−0.09	0.08	0.27 **	0.34 **
10	Gender	−0.14	0.21 *	−0.02	0.09	−0.06	−0.09	0.01	−0.21 *	1	−0.10	−0.05	0.02	0.07	−0.03	−0.04	−0.38 **
11	Age	0.08	−0.012	−0.15	−0.05	−0.09	0.11	−0.08	−0.05	−0.10	1	0.78 **	−0.09	0.14	0.17*	−0.09	0.16
12	Year	0.17 *	0.15	−0.09	0.02	−0.04	0.09	−0.06	0.00	−0.05	0.78 **	1	−0.15	0.30 **	0.27 **	0.14	0.22 **
13	Ethnicity	0.17 *	0.08	0.13	0.12	0.03	0.01	−0.01	−0.04	0.02	−0.09	−0.15	1	−0.04	−0.11	0.10	−0.06
14	Lab Courses	0.08	0.13	0.01	−0.02	0.16	−0.12	−0.11	−0.09	0.07	0.14	0.30 **	−0.04	1	0.17 *	0.09	0.04
15	Articles Read	0.20 *	0.23 **	0.17 *	0.15	0.22 **	−0.04	0.03	0.08	−0.03	0.17 *	0.27 **	−0.11	0.17 *	1	0.03	0.09
16	Letter Sets total score	0.21 *	0.24 **	0.28 **	0.18 *	0.36 **	0.06	0.16	0.27 **	−0.04	−0.09	0.14	0.1	0.09	0.03	1	0.37 **
17	Number Series	0.33 **	−0.00	0.04	0.08	0.17 *	0.21 *	0.20 *	0.34 **	−0.38 **	0.16	0.22 **	−0.06	0.04	0.09	0.37 **	1

** Correlation is significant at the 0.01 level (2-tailed). * Correlation is significant at the 0.05 level (2-tailed).

**Table 11:** Principal component analyses in Study 2 without new scales with analytical ability tests.

Rotated Component Matrix ^a^		
	Component	
	1	2
Generating Hypotheses total score averaged over both raters	0.75	0.05
Generating experiments total score averaged over both raters	0.77	0.01
Drawing conclusions total score averaged over both raters	0.66	0.32
Total Score Letter Sets	0.34	0.74
Number Series correct answers	−0.08	0.87

Extraction Method: Principal component analysis. Rotation Method: Varimax with Kaiser normalization. ^a^ Rotation converged in three iterations. Two principal components had Eigenvalues greater than 1. Component 1 had an Eigenvalue of 1.70, accounting for 34.1% of the variance in the data. Component 2 had an Eigenvalue of 1.41, accounting for 28.2% of the variance in the data. Cumulative percent variance accounted for was 62%.

**Table 12:** Principal component analyses in Study 2 without new scales, but with SAT.

Rotated Component Matrix ^a^		
	Component	
	1	2
Generating Hypotheses total score averaged over both raters	0.17	0.69
Generating experiments total score averaged over both raters	0.00	0.78
Drawing conclusions total score averaged over both raters	0.07	0.71
SAT Math Score	0.91	0.04
SAT Reading Score	0.90	0.16

Extraction Method: Principal component analysis. Rotation Method: Varimax with Kaiser normalization. ^a^ Rotation converged in three iterations. There were two principal components with Eigenvalues greater than 1. Component 1 had an Eigenvalue of 1.67, accounting for 33.5% of the variance in the data. Component 2 had an Eigenvalue of 1.61, accounting for 32.1% of the variance in the data. Cumulative percent variance accounted for was 66%.

**Table 13:** Principal component analyses in Study 2 with SAT and analytical ability tests.

Rotated Component Matrix ^a^			
	Component		
	1	2	3
Generating Hypotheses total score averaged over both raters	0.19	−0.01	0.79
Generating experiments total score averaged over both raters	0.01	0.13	0.75
Drawing conclusions total score averaged over both raters	−0.06	0.55	0.52
SAT Math Score	0.87	0.29	−0.03
SAT Reading Score	0.91	0.02	0.20
Total Score Letter Sets	0.12	0.77	0.20
NS correct answers	0.22	0.80	−0.07

Extraction Method: Principal component analysis. Rotation Method: Varimax with Kaiser normalization. ^a^ Rotation converged in five iterations. There were three components with Eigenvalues greater than 1. Component 1 had an Eigenvalue of 1.68, accounting for 24.0% of the variance in the data. Component 2 had an Eigenvalue of 1.64, accounting for 23.4% of the variance in the data. Component 3 had an Eigenvalue of 1.53, accounting for 21.9% of the variance in the data. Cumulative percent variance accounted for was 69%.

**Table 14:** Rotated maximum likelihood common factor analyses in Study 2 without new scales with analytical ability tests.

Rotated Factor Matrix ^a^		
	Factor	
	1	2
Generating Hypotheses total score averaged over both raters	0.18	0.48
Generating experiments total score averaged over both raters	0.03	0.68
Drawing conclusions total score averaged over both raters	0.25	0.52
Total Score Letter Sets	0.98	0.22
NS correct answers	0.36	0.09

Extraction Method: maximum likelihood common factor analysis. Rotation Method: Varimax with Kaiser normalization. ^a^ Rotation converged in three iterations. Two common factors had Eigenvalues greater than 1. Factor 1 had an Eigenvalue of 1.17, accounting for 23.5% of the variance in the data. Factor 2 had an Eigenvalue of 1.02, accounting for 20.4% of the variance in the data. Cumulative percent variance accounted for was 44%.

**Table 15:** Rotated maximum likelihood common factor analysis with SAT.

Rotated Factor Matrix ^a^		
	Factor	
	1	2
Generating Hypotheses total score averaged over both raters	0.17	0.50
Generating experiments total score averaged over both raters	0.06	0.62
Drawing conclusions total score averaged over both raters	0.03	0.51
SAT Math Score	0.66	0.08
SAT Reading Score	0.99	0.17

Extraction Method: Maximum likelihood common factor analysis. Rotation Method: Varimax with Kaiser normalization. ^a^ Rotation converged in three iterations. There were two (unrotated) factors with Eigenvalues greater than 1. After rotation, the second factor had an Eigenvalue less than 1. Factor 1 had an Eigenvalue of 1.45, accounting for 28.9% of the variance in the data. Factor 2 had an Eigenvalue of 0.93, accounting for 18.5% of the variance in the data. Cumulative percent variance accounted for was 47%.

**Table 16:** Rotated maximum likelihood common factor analysis with sat and analytical ability tests.

Rotated Factor Matrix ^a^			
	Factor		
	1	2	3
Generating Hypotheses total score averaged over both raters	0.12	0.07	0.59
Generating experiments total score averaged over both raters	0.05	0.10	0.52
Drawing conclusions total score averaged over both raters	0.00	0.39	0.45
SAT Math Score	0.68	0.37	0.00
SAT Reading Score	0.97	0.02	0.23
Total Score Letter Sets	0.09	0.64	0.27
NS correct answers	0.18	0.60	0.05

Extraction Method: Maximum likelihood common factor analysis. Rotation Method: Varimax with Kaiser normalization. ^a^ Rotation converged in six iterations. There were three unrotated factors with Eigenvalues greater than 1. When rotated, only two factors had Eigenvalues greater than 1. Factor 1 had an Eigenvalue of 1.46, accounting for 20.9% of the variance in the data. Factor 2 had an Eigenvalue of 1.08, accounting for 15.4% of the variance in the data. Factor 3 had an Eigenvalue of 0.95, accounting for 13.5% of the variance in the data. Cumulative percent variance accounted for was 50%.

**Table 17:** Rotated principal component matrix in the Study 2 analysis of the total scores of all our subscales (including editor and reviewer items) and the analytical ability tests.

Rotated Component Matrix ^a^		
	Component	
	1	2
Editor Items	0.30	0.55
Reviewer Items	0.73	0.06
Generating Hypotheses	0.78	0.01
Drawing conclusions	0.46	0.41
Generating experiments	0.62	0.23
Average SAT Score (math and reading)	0.02	0.67
Average analytical ability scores (LS and NS)	0.06	0.82

Extraction Method: principal component analysis. Rotation Method: Varimax with Kaiser normalization. ^a^ Rotation converged in three iterations. There were two principal components with Eigenvalues greater than 1. Component 1 had an Eigenvalue of 1.83, accounting for 26.1% of the variance in the data. Component 2 had an Eigenvalue of 1.64, accounting for 23.5% of the variance in the data. Cumulative percent variance accounted for was 50%.

**Table 18:** Rotated principal component matrix in the Study 2 analysis of the total scores of all our subscales with the exception of the editor items, and the analytical ability tests and SAT.

Rotated Component Matrix ^a^		
	Component	
	1	2
Reviewer Items	0.75	−0.00
Generating Hypotheses	0.78	0.10
Drawing conclusions	0.45	0.49
Generating experiments	0.61	0.20
Average SAT Score (math and reading)	0.04	0.74
Average analytical ability scores (LS and NS)	0.12	0.82

Extraction Method: Principal component analysis. Rotation Method: Varimax with Kaiser normalization. ^a^ Rotation converged in three iterations. There were two principal components with Eigenvalues greater than 1. Component 1 had an Eigenvalue of 1.75, accounting for 29.2% of the variance in the data. Component 2 had an Eigenvalue of 1.50, accounting for 25.0% of the variance in the data. Cumulative percent variance accounted for was 54%.

**Table 19:** Rotated factor matrix in the Study 2 maximum likelihood factor analysis of the total scores of all our subscales (including editor and reviewer items) and the analytical ability tests.

Rotated Factor Matrix ^a^		
	Factor	
	1	2
Editor Items	0.36	0.30
Reviewer Items	0.11	0.58
Generating Hypotheses	0.11	0.62
Drawing conclusions	0.35	0.35
Generating experiments	0.23	0.45
Average SAT Score (math and reading)	0.35	0.15
Average analytical ability scores (LS and NS)	0.86	0.04

Extraction Method: Maximum likelihood common factor analysis. Rotation Method: Varimax with Kaiser normalization. ^a^ Rotation converged in three iterations. There were two common factors with Eigenvalues greater than 1. Factor 1 had an Eigenvalue of 1.18, accounting for 16.8% of the variance in the data. Factor 2 had an Eigenvalue of 1.16, accounting for 16.6% of the variance in the data. Cumulative percent variance accounted for was 33%.

**Table 20:** Rotated factor matrix in the Study 2 maximum likelihood factor analysis of the total scores of all our subscales with exception of the editor items, and the analytical ability tests and SAT.

Rotated Factor Matrix ^a^		
	Factor	
	1	2
Reviewer Items	0.12	0.52
Generating Hypotheses	0.12	0.73
Drawing conclusions	0.34	0.35
Generating experiments	0.17	0.42
Average SAT Score (math and reading)	0.33	0.19
Average analytical ability scores (LS and NS)	0.97	0.07

Extraction Method: Maximum likelihood common factor analysis. Rotation Method: Varimax with Kaiser normalization. ^a^ Rotation converged in three iterations. There were two common factors with Eigenvalues greater than 1. Factor 1 had an Eigenvalue of 1.21, accounting for 20.2% of the variance in the data. Factor 2 had an Eigenvalue of 1.14, accounting for 19.0% of the variance in the data. Cumulative percent variance accounted for was 39%.

## Appendix A. Generating Hypotheses

On the next page, you will read several scenarios that describe a situation as well as a hypothesis to explain the facts presented. You will be asked to think of some other explanations (alternative hypotheses) that can explain the circumstances presented. Please write down any alternative explanations you can think of. There is no time limit for this exercise.

### A.1. Example

Eve is interested in studying the effects of taking exams on student performance. She devises an experiment where group A students are given weekly quizzes and bi-semester exams, while group B students are only given bi-semester exams. The results show that students in group A do better overall than do students in group B. She explains that weekly quizzes help the students stay on track with the material.

What are some alternative hypotheses regarding why the students who receive weekly quizzes perform better than the students who don't?

### A.2. Potential Answers

- It may be that the quizzes allow the students in group A to make mistakes and learn from them before taking the exams.
- It may be that the students in group A are simply better exam takers than students in group B, regardless of any prior exams they may have taken.
- It may be that students in group B are not used to the types of questions that Eve tends to ask on the quizzes/exams.
- It may be that there are more students who are good at math in group A than group B, which skews the data.
- It may be that Group A was exposed to other variables (e.g., a more effective teacher) which would have resulted in higher scores than group B, even if they were not taking weekly exams.
- It may be that the exams are biased towards students in Group A, say by asking the same questions that were already assessed in the quizzes.

1. Marie is interested in child development. One day, she notices that whenever Laura’s nanny comes in to pick up Laura from nursery school, Laura starts to cry. Marie reflects upon how sad it is that Laura has a poor relationship with her nanny.

What are some alternative hypotheses regarding why Laura starts to cry when she is picked up from nursery school by the nanny?

2. Jane is interested in the relationship between HIV/AIDS illness and depression. In one study, she finds that 10% of subjects without HIV/AIDS are clinically depressed, whereas 60% of subjects with HIV/AIDS are clinically depressed. Upon consideration of the data, Jane hypothesizes that subjects with HIV/AIDS are more likely to develop clinical depression because they are aware of their critical condition and often feel hopeless about it.

What are some alternative hypotheses regarding why subjects with HIV/AIDS are more likely to develop clinical depression?

3. In his bestselling book *Freakonomics*, economist Steve Levitt suggested that the crime rate in the US declined dramatically in the early 1990s because many women, whom he described as “young, single, poor, less affluent, and not ready to raise children,” underwent abortions. He claimed that children born into challenging circumstances such as these are more likely to commit a crime later on in life.

**What are some alternative hypotheses regarding why the crime rate plummeted in the early 1990s?**

## Appendix B. Generating Experiments

On the next page, you will read several scenarios that describe a situation as well as a hypothesis. You will be asked to design an experiment for each of those scenarios to test the hypothesis presented. There is no time limit for this exercise. (Type 2)

### B.1. Note

You do not need to be familiar with specific tests in any of the subject areas presented. For example, if you want to assess stereotypes toward a target group and are not familiar with tests that assess stereotypes, just write in your answer that a test assessing stereotypes toward a particular target group should be administered.

### B.2. Here is an Example

Martin believes that a particular yellow food dye (E104) not only causes hyperactivity in children (as has been proven), but also increases people’s divergent thinking. That is, he believes this dye puts people in a state in which they are more creative. How can he test his hypothesis that the dye E104 increases creativity?

### B.3. Possible Answer

Recruit 100 participants. Give half of them a beverage that contains E104, and half of them a similar beverage without that dye. After half an hour, assess their creativity by means of several tests like the Torrance test of divergent thinking, the alternative uses test by Guilford, or the cartoon caption test by Sternberg.

1. Ella, a senior in college, observes that her roommate tends to perform better on an exam if she has had a cup of coffee beforehand. Ella hypothesizes that drinking coffee before taking an exam will significantly increase one's exam performance. However, Ella does not know how to test this hypothesis.

Please suggest an experimental design to test this hypothesis and describe the experiment in some detail. Assume you have the resources you need to be able to do the experiment (e.g., access to students and their academic records, sufficient funds to pay subjects, etc.).

2. John hypothesizes that his brother’s playing of violent video games has increased his brother's aggressive behavior. John is not sure, however, whether playing violent video games really increases aggression.

Please suggest an experimental design to test John’s hypothesis and describe the experiment in some detail. Assume you have the resources you need to be able to do the experiment (e.g., access to violent video games, subjects, sufficient funds to pay subjects, etc.).

3. Ariel, a teacher, has noticed that her students seem to pay better attention in the classroom after playing an outdoor sport. Ariel hypothesizes that the students will focus better in the classroom after playing in an outdoor sport. However, Ariel does not know how to test her hypothesis.

Please suggest an experimental design to test this hypothesis and describe the experiment in some detail. Assume you have the resources you need to be able to do the experiment (e.g., access to subjects, sufficient funds to pay subjects, etc.).

## Appendix C. Drawing Conclusions

On the next page, you will read several scenarios that describe an experiment that was conducted to test a specific hypothesis. However, each one of these experiments is flawed in some way. You will be asked to consider the experimental design and point out the flaws. Please write down any flaws you can come up with (one is enough but if you can think of more, then please write them down as well). There is no time limit for this exercise.

### C.1. Example

We tested the hypothesis that when a salesperson smiles directly at a customer, the individual is more likely to make a sale than when the salesperson fails to smile. Five saleswomen at a bridal shop were instructed to do one of three things while trying to sell a wedding dress to a customer: either to smile directly (in the face of) the customer, smile indirectly (while looking away from) the customer, or have a neutral expression on the face. It was found that smiling directly at customers did indeed increase sales significantly. Fewest wedding dresses were bought in the indirect-smiling condition. It was concluded that salespeople should smile directly into the faces of their customers if they wish to increase their sales effectiveness.

**Is this conclusion correct? Why or why not?**

### C.2. Possible Answer

The conclusion is not correct because:

1. All customers were women so one cannot generalize to all customers.
2. All salespeople were women so one cannot generalize to all salespeople.
3. Bridal-shop customers are not representative of customers in general.
4. The conclusion that looking away from customers (indirect smiling condition) was crucial in producing the lowest sales would not follow conclusively unless there were two clear conditions in which the salesperson had a neutral expression, either looking directly at the customer or looking away from the customer.

1. Bill was interested in how well a new program for improving mathematical performance worked. He gave 200 students a pretest on their mathematical knowledge and skills. He then administered the new program to them. After administering the program, he gave the same 200 students a posttest that was equal in difficulty and in all relevant ways comparable to the pretest. He found that students improved significantly in performance from pretest to posttest. He concluded that the program for improving mathematical performance was effective.

**Is this conclusion correct? Why or why not?**

2. Mary believed that administering 400 mg of Vitamin C two hours before a test would improve performance on the test. She randomly assigned 200 subjects either to Group A or Group B. She administered 400 mg of Vitamin C to Group A subjects and then gave them a test two hours later. She also had a control group, Group B, to which she administered nothing at all. She just gave them the same test Group A had gotten. She found that Group A performed at a higher level than did Group B. She concluded that 400 mg of Vitamin C administered two hours before the test improved subjects’ performance.

**Is this conclusion correct? Why or why not?**

3. Anthony wanted to see whether eating breakfast improves performance on tests administered during the first period of the day in high school. He had 10 subjects eat breakfast and 10 not eat breakfast before a first-period test. He failed to find a significant difference between those who ate breakfast and those who did not. He concluded that eating breakfast did not result in better performance on tests during the first period of the day in high school.

**Is this conclusion correct? Why or why not?**

## Appendix D. Reviewer Items

On the next page, you will read a short research article. We will then ask you to play the role of a reviewer and point out any flaws and problems you can find with the article. Here is a short example (in our example, you will be presented just with the abstract of an article):

We tested subjects to determine whether people remember concrete nouns (e.g., ball) better than abstract nouns (e.g., truth). A sample of 20 college-student subjects was divided at random into two groups of 10 persons each. One group was given 30 min to memorize a list of abstract nouns; the other group was given 25 min to memorize a list of concrete nouns. At the end of the memorization time period, all subjects were tested on free recall of the nouns they had memorized. As predicted, students learning the concrete nouns performed better than students learning the abstract nouns, although the difference was not statistically significant.

### D.1. Review of Abstract

This abstract is not fully adequate:

- The abstract does not tell the relative number of men and women, either in total or in the two groups.
- We learn nothing of the ethnicity or general location of the students, or of the kind of college they attended.
- The two groups were not given equal time to memorize their respective lists, a major flaw.
- Because the difference was not statistically significant, the author should not say that the students in the concrete-noun group performed better. In fact, the difference was statistically meaningless.

### D.2. Short Report

**A Comparison of Intelligence Levels among People Living in Rural, Suburban, and Urban Environments**

**Mike Mason**

University of the Middle West

Our goal in this study was to compare the relative intelligence of people growing up in rural, suburban, and urban areas. In other words, does the kind of environment in which one grows up determine with 100% certainty one’s intelligence. The work is based on Edwin Boring’s (1923) proven theory that intelligence is whatever intelligence tests measure, nothing more, nothing less. Because it is not clear which kind of environment would produce the highest level of intelligence, there were no a priori hypotheses in this study about factors affecting intelligence or how they would affect it; rather, the data would show what is true.

#### D.2.1. Methods

##### Subjects

The subjects in this experiment were 30 individuals. All of them were adults—that is, 18 years or older, and most were males. One-third of them came from rural environments, one-third from suburban environments, and one-third from urban environments. All of the subjects were from a small college large Midwestern state. In all, 1/3 of the subjects were college educated or educated beyond college. Also, those with graduate degrees should be separated from those with just undergraduate degrees. Ideally, those with doctorates would be separated from those with masters. Subjects were mostly from European-American backgrounds, reflecting the population of the state. All subjects gave informed consent to participating in the study. At the end of the study, subjects who were interested received oral debriefing. Data from five subjects were eliminated either because they did not complete the demographic questionnaire or because their responses on the intelligence test indicated that they were not making a serious effort to complete the items in a conscientious manner

##### Materials

There were two kinds of materials in the study: a demographic questionnaire and a nonverbal intelligence test, the Cattell Culture-Fair Test of g, Level 2. The demographic questionnaire asked about age, sex, and current living arrangement (rural, suburban, urban). Subjects were asked simply to check whether their residence was rural, suburban, or urban. The intelligence test contained four subtests requiring reasoning with geometric shapes; all measured so-called “fluid” intelligence. In order to fit the session into the 50 min allocated for the experiment, both the demographic questionnaire and the intelligence test were timed. Recommended test time limits had to be slightly shortened to fit into the allowable time interval. The IQ test was scored for number correct across the four subtests. There was no penalty for guessing.

##### Design

The main independent variable in the study was living situation (rural, suburban, urban). Other independent variables were age, sex, and ethnicity. The main dependent variable was total raw score converted to an IQ on the intelligence test, using a single set of adult norms. Subjects were assigned randomly to groups.

##### Procedure

Subjects entered the room, took a seat, and filled out an informed-consent form. Then subjects completed the demographic questionnaire. They were allotted 30 s to do so. Then subjects completed the four tests of the Cattell test, in order. At the end, subjects who wished received oral debriefing. Subjects were paid as they left the room.

#### D.2.2. Results

Mean IQs were 97.9 for the rural group, 100.2 for the suburban group, and 101.5 for the urban group. Data were analyzed via a one-way analysis of variance. The difference among groups was not statistically significant, *F* (3, 60) = 1.42. There were no effects of sex of subjects. It was concluded that living arrangement—rural, suburban, urban—has no effect on IQ.

#### D.2.3. Discussion

The results were, of course, disappointing. On the positive side, these results indicate that parents do not have to worry about whether they bring up their children in a rural, suburban, or urban environment: The children will be equally intelligent as adults regardless of type of environment. It will be interesting to see whether the same results we obtained for adults also hold for children. We also would like to see whether similar results would be obtained in a predominantly Black population. In the future, we also would like to use larger samples. Also, a weakness of our study was that all the adults were from the same state. However, we hasten to point out that ours was a very typical Midwestern state. An advantage of our study is that we used a highly regarded and reputable test of intelligence. Also, it is worth noting that several subjects commented favorably on our study as they left the room. Finally, we hope that others will follow our novel lead and test relative levels of intelligence in different kinds of groups.

**Is the article you just read adequate or not? If not, what are the article’s flaws and problems? (Participants answer in essay style)**

### D.3. Short Report

**A Validation of the Johnson Extraversion Scale, Forms 1 and 2**

**Jason Johnson**

University of the Middle West

The purpose of this study was to validate two alternate forms of the Johnson Extraversion Scale, a scale created by the author of this article. The purpose the scale is to provide two alternate forms of a scale that measures extraversion. Extraversion can be defined as people’s tendency to be likable to others. Extraversion is important because it is one of the scales in the five-factor theory of personality. People who are extraverted are more likely to succeed in a variety of occupations, such as salesperson, manager, teacher, or lawyer, among many others. Also, research shows that extraverted people are more likable. Because existing scales of extraversion are inadequate for a variety of reasons, and often impractical to administer, the two new scales will be welcomed by researchers and practitioners alike. Sometimes, researchers and practitioners need two different but comparable forms of an extraversion scale, such as before and after an intervention. Some researchers may give the same scale twice, but giving the same scale twice is generally a bad idea because of the possibility of practice effects—that is, subjects do better the second time simply because they already have taken the test twice. Thus, the research here is useful because it provides a scale measuring social competence through two different forms of the same test.

#### D.3.1. Methods

##### Subjects

Subjects in the study were 200 undergraduates from a large Middle Western university. In all, 95 subjects were females and 105 subjects were males. Subjects either volunteered for course credit (Form 1 of the test) or else were paid $15 for participation (Form 2 of the test). Subjects all were between 18 and 20 years of age. Subjects were not asked questions about race or socioeconomic status because of the sensitivity of these issues.

##### Materials

There were two forms of the Johnson Extraversion Scale. Form 1 of the scale, the longer form, had 50 items, and Form 2, the shorter form, had 25 items. All items were in the form of true-false statements to be rated by subjects taking the scale. Two examples of statements were “I like to be with other people” (Form 1) and its parallel item “I like being with other people” (Form 2). Scoring was based on the number of statements rated as “true,” with higher scores indicating a higher level of extraversion. Subjects also took another test of extraversion, taken from the Internet, to ensure that our scale measured the same thing as other scales measuring extraversion.

##### Design

Subjects all took both Form 1 and Form 2 as well as the Internet-based test. In particular, those who randomly signed up for the earlier session were given Form 1 first and those who signed up for the later session were given Form 2 first. The design was a correlational one.

##### Procedure

Subjects came into the testing room. They took a first form of the Johnson Test, then a second form, then the Internet-based test. They had 50 min to complete all three tests. Subjects were told that the measure was true-false, and that they should indicate for each item whether it was true or false. Subjects were also told that there were no right or wrong answers, only better or worse answers. All subjects were debriefed at the end of the study, at which point it was explained to them that our goal was to create tests that would measure their ability to get along with other people. If subjects had questions beyond the debriefing, they were told to get in touch with the experimenter after all subjects in the study had been tested.

#### D.3.2. Results

The results indicated that the two forms were reliable, with reliabilities of 0.74 for Form 1 and 0.68 for Form 2. There were no sex differences. The correlation between the two forms was 0.66, indicating that the forms were roughly parallel. The correlations with the Internet-based test were 0.41 for Form 1 and 0.38 for Form 2, suggesting that the Internet-based test was no, after all, a particularly strong measure of extraversion. It was concluded that the new assessments provide reliable and valid measures of extroversion in the adult population

#### D.3.3. Discussion

The results suggest the extraversion is indeed an important attribute of personality to measure. The two forms of the test may contribute to measurement of extraversion by providing two different parallel assessments, a longer and shorter form, that researchers and practitioners can now use. Further research will look at whether the tests correlate with intelligence. Our goal ultimately is to understand the nature of extraversion. We already are testing other populations to see how they will score on our scale.

**Is the article you just read adequate or not? If so, what are the article’s flaws and problems? (Participants answer in essay style)**

## Appendix E. Editor Item

On the next page, you will read a short review of an article submitted for publication. We will then ask you to play the role of a journal editor (we are keeping in mind that you have not read the original article on which the review is based):

- In what ways is the review useful?
- In what ways is it not useful?
- How could it have been better?
- What did the referee do wrong and what should the referee do differently in the future?
- Note: You do NOT have to comment on each sentence.

**Excerpt from a Review of an Article Submitted to a Journal**

1. This article is simply awful.
  This first sentence is unnecessarily hostile.
2. The author did not think before submitting it for publication.
  The second sentence is ad hominem—an attack on the author rather than on the substance of the article.
3. Although it claims to be about memory in general, it really only deals with recall memory.
  The third point is valid, and should be addressed in a revision of the article.
4. Also, the numbers of subjects in the two groups are not equal.
  The author does not say why having unequal numbers in the two groups is problematical.
5. Furthermore, the statistical tests performed are inadequate to testing they hypotheses.
  The reviewer should have said why the statistical tests are inadequate.

Review of Article Submitted to *Journal of Development*

1. The article “Attachment Styles in Children of Middle Childhood,” explores the various attachments styles found in children of 8 to 12 years old, including secure, anxious-resistant, anxious-avoidant, and disorganized/disoriented. The article finds that distributions of attachment styles actually change with age.
  (Participants comment on each item individually in essay style)
2. The authors of the article show themselves to be foolish.
3. For one thing, their whole study is suspect because they do not have exactly equal numbers of children of 8, 9, 10, 11, and 12 years of age. For example, there are 198 8-year-olds but 203 10-year-olds.
4. For another thing, they state the number of boys in the study (495) and the total number of subjects (1028), but never directly state the number of girls.
5. Another problem with the study is that the authors did not randomly assign students either to age groups or to attachment styles, so random assignment, the gold standard for experimental research, was lacking.
6. The study was conducted only with subjects in the Northeast, so it is not clear whether the results would generalize to other parts of the country or to other countries.
7. In the Results section, a power analysis has been done, but what really needs to be done is an analysis to understand whether the sample sizes were large enough to detect whether there were effects of a reasonable size and significance level.
8. A number of effects are significant at the 0.001 level, but what we do not know from the data analysis is whether these effects were also significant at the standard 0.05 level of significance.
9. Curiously, the article never really defines what the different attachment styles are so it is more difficult than it should be to interpret the results.
10. We also cannot determine whether these results would generalize to people later in their lives, such as when they are in their old age.
11. Nowhere in the study are my own studies on attachment cited, which shows a distinct lack of scholarship.
12. The writing in the study also was hard to understand because of overuse of abbreviations that were never defined.
13. Finally, I cannot recommend this study for publication because the results were not shown to be the same for boys and girls, suggesting a bias against girls in the study.
